# Using Community Engagement and Geographic Information Systems to Address COVID-19 Vaccination Disparities

**DOI:** 10.3390/tropicalmed7080177

**Published:** 2022-08-11

**Authors:** Tsu-Yin Wu, Xining Yang, Sarah Lally, Alice Jo Rainville, Olivia Ford, Rachel Bessire, Jessica Donnelly

**Affiliations:** 1 Center for Health Disparities Innovations and Studies, Eastern Michigan University, Ypsilanti, MI 48197, USA; 2 Geography and Geology, Eastern Michigan University, Ypsilanti, MI 48197, USA; 3 Human Nutrition, Eastern Michigan University, Ypsilanti, MI 48197, USA; 4 Dietetics and Human Nutrition, Eastern Michigan University, Ypsilanti, MI 48197, USA

**Keywords:** SARS-CoV-2, vaccine access, Asian Americans, communities of color, minority, geographic information system (GIS)

## Abstract

The COVID-19 pandemic has exacerbated existing health disparities and had a disproportionate impact on racial and ethnic minority groups in the United States. Limited COVID-19 data for Asian Americans have led to less attention for this population; nevertheless, available statistics have revealed lesser known impacts of COVID-19 on this population. Even with significant increases in vaccine supply and recent increases in COVID-19 vaccination rates, racial and ethnic disparities in vaccine uptake still persist. These disparities are amplified for individuals with limited English proficiency (LEP). The purpose of this paper is to apply community-engaged and geographic information system (GIS) strategies to increase equitable access to COVID-19 vaccination uptake by decreasing the structural barriers to COVID-19 vaccine uptake, with a particular focus on Asian Americans with LEP. Building upon existing community-academic partnerships between the academic unit and community-based organizations, the project team established community-led mobile and pop-up COVID-19 vaccination clinics to reach underserved individuals in their communities, worked with commercial pharmacies and reserved appointments for community-based organizations, used GIS to establish COVID-19 vaccination sites close to communities with the greatest need, and deployed trusted messengers to deliver linguistically and culturally relevant COVID-19 vaccine messages which built vaccine confidence among the community members. The implementation of mobile clinics expanded COVID-19 vaccine access and community-driven, multi-sector partnerships can increase the capacity to enhance efforts and facilitate access to COVID-19 vaccination for hard-to-reach populations.

## 1. Introduction

The COVID-19 pandemic has exacerbated existing health disparities and had a disproportionate impact on racial and ethnic minority groups in the United States [1]. Limited COVID-19 data for Asian Americans potentially led to less attention for this population; nevertheless, available statistics have revealed lesser known impacts of COVID-19 on this population. The Morbidity and Mortality Weekly Report showed that Asian Americans (AAs) had the second highest percentage increase in excess deaths in the first seven months of 2020 [2]. About 30% of AAs are part of the essential workforce, which experiences greater risk of exposure [3]. Additionally, AAs are more likely to live in multigenerational homes than other racial/ethnic groups [4]. Crowded housing has been linked to the spread of diseases and psychological distress. In some states, AAs make up the highest share of healthcare workers with a large proportion of these individuals being immigrants [5]. Approximately one third of AAs have limited English proficiency, which is much higher than the overall population (8.2%) in the United States. This creates additional structural barriers for AAs with respect to COVID-19 testing and vaccination [6].

Asian Americans are the fastest-growing minority group in the United States. In Michigan (MI), Asians represent 3.4% of the state’s population [7], albeit a smaller percentage compared to other larger states but it is steadily growing. Asian Indians, Chinese, Korean, Japanese, and Filipino are the largest Asian subgroups living in MI [7]; however, other Asian subgroups, including Vietnamese, Thai, Nepalese, Hmong, and Burmese are increasing as immigrants arrive.

While the vaccine supply increased significantly in 2021 with recent increases in COVID-19 vaccination rates, racial and ethnic disparities in vaccine uptake still persist [8]. These disparities are amplified for individuals with limited English proficiency (LEP), who often experience additional barriers to healthcare access related to language, culture, and systematic influences. Therefore, it is imperative to explore strategies to achieve equity in access to and administration of COVID-19 vaccination among racial and ethnic minority groups who have been disproportionately affected by COVID-19 [9,10], such as individuals with LEP [11,12].

The purpose of this article is to apply community-engaged geographic information system (GIS) strategies to increase equitable access for COVID-19 vaccination uptake by decreasing the structural barriers to COVID-19 vaccination with a particular focus on AAs with LEP in West and Southeast Michigan. Building upon existing community-academic partnerships between the academic unit and community-based organizations, the Eastern Michigan University Center for Health Disparities Innovations and Studies (EMU CHDIS) addressed multilevel barriers to vaccination among AAs with LEP, provided assistance to bridge the digital divide of individual technological proficiency, mitigated vaccine hesitancy, and ultimately, improved vaccine equity. The following strategies were implemented to decrease barriers and build trust in COVID-19 vaccines among underserved Asian American (AA) populations in Michigan.

## 2. Community Engagement: Establishing Community-Led Mobile and Pop-Up COVID-19 Vaccination Clinics to Reach People in Their Communities

A needs assessment was conducted by the project team regarding access to COVID-19 vaccinations. Four hundred and forty-two AAs in Michigan completed the survey. Respondents were recruited with the help of community partners through social media, ethnic newspapers, and personal connections. Surveys were administered at community centers, mosques, temples, churches, and a nail salon. Respondents self-reported race/ethnicity, which was later categorized into South Asian (Asian Indian, Bangladeshi, Burmese), Southeast Asian (Filipino, Korean, Vietnamese), East Asian (Chinese, Japanese, Taiwanese), and Non-Asian. Additional demographic questions and questions assessing perceptions of COVID-19 and COVID-19 vaccines and access to COVID-19 vaccinations were also included. Surveys were available in English and seven Asian languages (Chinese, Korean, Bangla, Thai, Tamil, Urdu, and Vietnamese).

The results from the needs assessment showed that more than 80% of these individuals reported having LEP. In addition, while more than 70% reported they had access to the internet and could use the internet, 59% stated that they did not know how to use the internet to register for COVID-19 vaccines/boosters and 60% did not know how to find places to get COVID-19 vaccinations.

Partnering with community-based organizations, CHDIS established 34 COVID-19 vaccination sites in 2021 at trusted locations where individuals and families regularly visit including mosques, churches, grocery stores, and community centers. Thus, increasing access to COVID-19 vaccines for this population. Our needs assessment revealed AAs with LEP had difficulty registering for and/or scheduling appointments at mass COVID-19 vaccination sites and pharmacy- or hospital-based sites. As a result, CHDIS mobile COVID-19 vaccination sites better served priority populations. Increasing vaccination sites that are at trusted locations and/or organized by trusted members of the community is a key component to addressing disparities in access for historically marginalized populations [13,14].

## 3. Bridging the Digital Divide: Partnered with Commercial Pharmacies and Reserved Appointments for Community-Based Organizations (CBOs)

The results of our needs assessment brought to light a common scenario in which AAs with LEP had access to the internet and computers but were unable to use these for scheduling COVID-19 vaccinations. While Michigan implemented vaccine locators and eligibility tools to inform the public about accessing pre-registration sites and/or hotline platforms, there appeared to be significant access barriers among historically marginalized populations; e.g., AAs with LEP. CHDIS secured appointments for CBOs who were then able to make appointments on behalf of community members and reduce barriers to registration.

## 4. Geographic Information System (GIS) Application: Using GIS to Establish COVID-19 Vaccination Sites Close to Communities with the Greatest Need

GIS technology has been used in previous vaccination research for planning vaccination campaigns, identifying populations that have not been vaccinated, and assessing the progress of vaccination campaigns [15,16,17,18]. As EMU CHDIS began planning and implementing mobile COVID-19 vaccination clinics for underserved AA communities in Michigan, the project team used spatial analysis in optimizing the location of vaccination sites and the distribution of vaccines. Specifically, we applied GIS mapping to assess the aforementioned needs geographically to ensure we were setting up vaccination sites in underserved neighborhoods. Sites of vaccination clinics included community centers, places of worship, Asian markets, and federally qualified health centers where community members gathered and/or received healthcare services. Using EMU CHDIS vaccine maps, the project team gained insights about variation in vaccine distribution and coverage metrics among priority populations in the locations where AAs reside, which helped the project team and EMU CHDIS community partners develop strategic vaccine distribution plans.

### 4.1. Data and Methods

The project team focused on the state of Michigan as the geographic area and used the counties and census tracts in the state of Michigan as the units of the mapping project. The county boundaries and census tracts GIS data were downloaded from the U.S. Census TIGER Geodatabases [19]. In addition to the GIS boundaries data, we identified the following variables as vaccine determinant layers in our GIS project: COVID-19 vaccination locations in the United States crowdsourced from URISA’s GISCorps [20], Michigan vaccination rates from Michigan COVID-19 Dashboard [21], the AA population, populations with no health insurance, and populations with no internet access by census tract from U.S. Census Bureau American Community Survey 5-year data (2016–2020) [22], and Michigan Social Vulnerability Index by census tract from CDC [23]. These variables were joined with the census tract or county GIS data to support the overlay analysis in the project. In addition, we geocoded all of the EMU CHDIS mobile clinics using the address information and converted the clinic events table into GIS point vector data, with the accurate latitude and longitude geographic coordinates for each event. All the data were managed using ESRI ArcGIS Pro software [24].

To identify the EMU CHDIS mobile clinic distribution in the state of Michigan, we first used the graduated symbols to visualize the mobile clinic events on the map. The size of the circle is in proportion to the number of vaccines administered at each location. Then a choropleth map was created for each vaccination determinant variable, in which a group of graduated colors was applied to correspond with an aggregate summary of a geographic characteristic within a census tract or county. To make the maps accessible to the public, the GIS project was published and shared on the ArcGIS Online platform, where the general public could see the maps via a URL link.

### 4.2. GIS Solutions

As part of the process, the project team deployed GIS to tabulate, visualize, and monitor key vaccine metrics and trends that supported the COVID-19 vaccination distribution plan. In the current phase of development, a web map [25] was launched including the locations, number of vaccines administered, type of vaccine, community partner, and dates of EMU CHDIS mobile clinic events. The vaccine maps also incorporated several key vaccine determinant layers which included AA populations, vaccine completion rate, computer accessibility, insurance coverage, and CDC Social Vulnerability Index (SVI). Constructed with census data at various scales of aggregation, CDC’s SVI classifies the relative social vulnerability of a location based on a combination of factors including socioeconomic status, household composition, disability status, minority status, language, housing type, and transportation [26]. EMU CHDIS incorporated these key determinant factors into geospatial data which assisted the project team in identifying pockets of AA populations to prioritize in our planning of mobile clinics to fill the gap. In the process, EMU CHDIS produced six maps.

**Figure 1:**
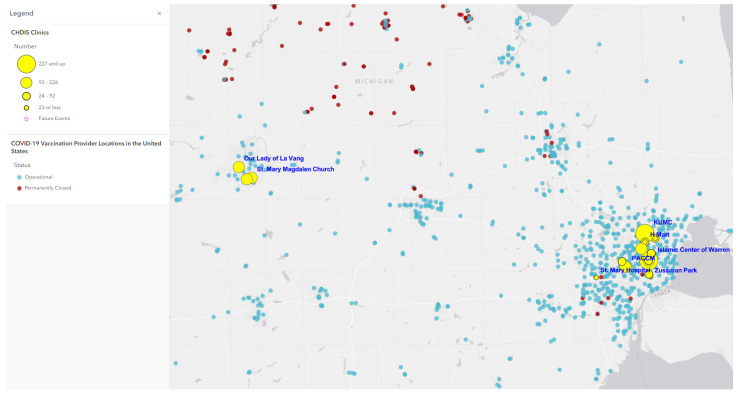
EMU CHDIS mobile clinics vs. COVID-19 vaccination provider locations.

**Figure 2:**
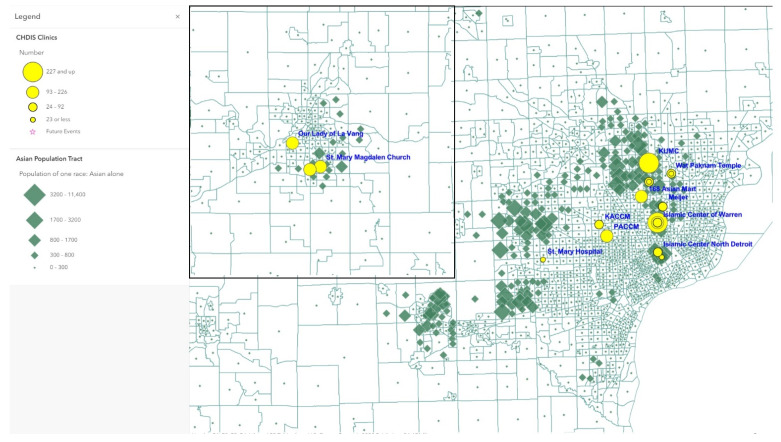
EMU CHDIS mobile clinics vs. Asian American populations.

**Figure 3:**
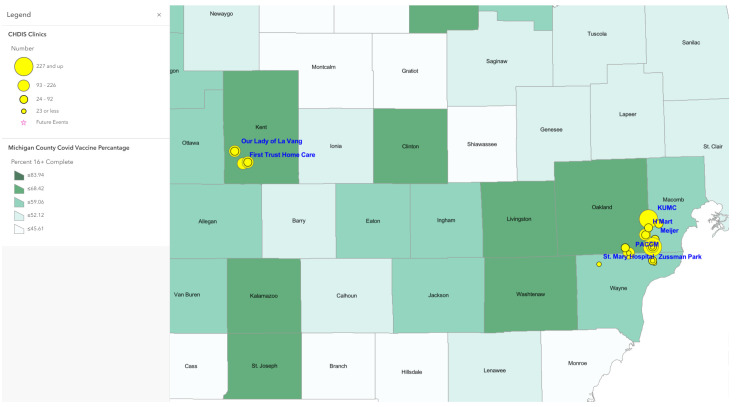
EMU mobile clinics vs. Michigan overall population COVID-19 vaccine completion percentage by county.

**Figure 4:**
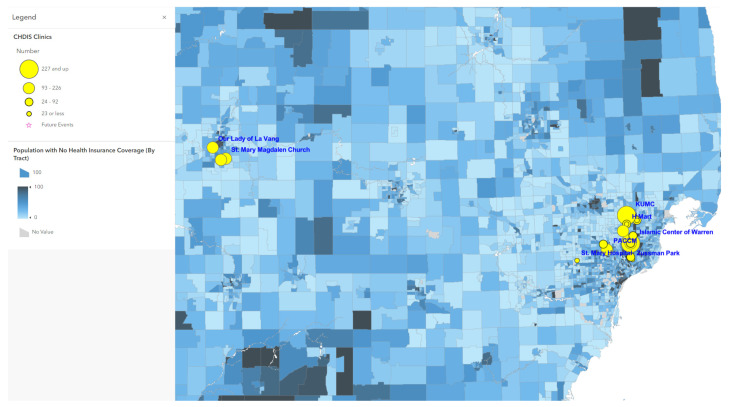
EMU mobile clinics vs. percent of population with no health insurance coverage.

**Figure 5:**
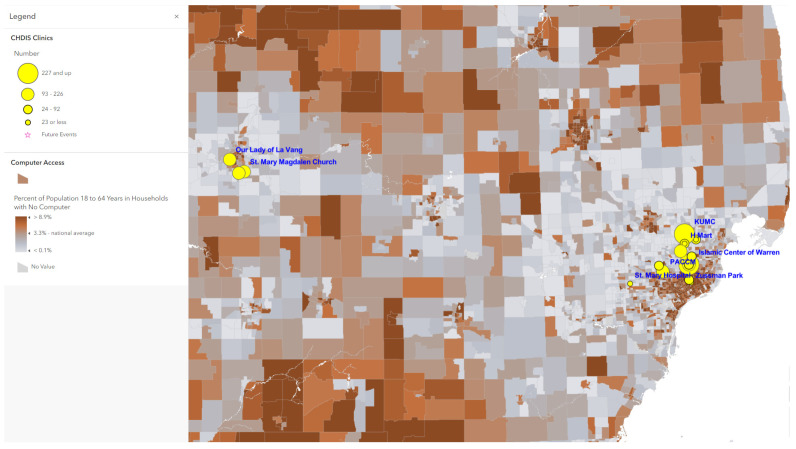
EMU mobile clinics vs. percent of population 18 to 64 years in households with no computer.

**Figure 6:**
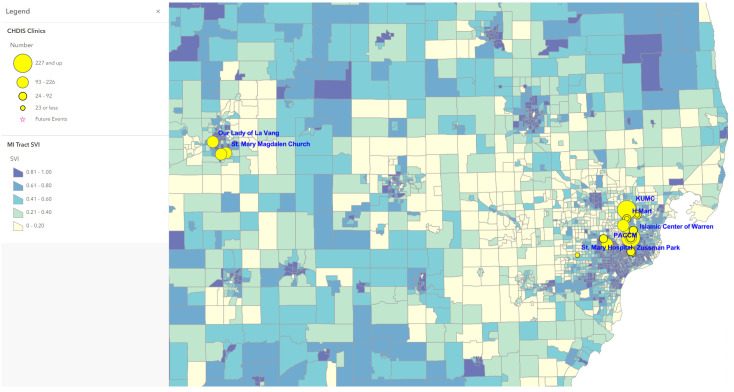
EMU mobile clinics vs. CDC/ATSDR Social Vulnerability Index in Michigan Census Tracts.

Map 1 (Figure 1) used ArcGIS Online to show a total of 41 CHDIS mobile clinics as of May 2022 in geographic locations (symbolized in yellow circles); these community-based clinics improved the coverage of vaccine opportunities based on the existing COVID-19 vaccination provider locations. Map 2 (Figure 2) used the graduated symbols of the AA population in each census tract to demonstrate that EMU CHDIS mobile clinics have successfully reached the priority population, AA, in areas where the concentration of AA population is medium-to-high. Map 3 (Figure 3) presented the overall population COVID-19 vaccine uptake percentage by county in Michigan and showed that EMU CHDIS mobile clinics played a role in narrowing the vaccine disparity gap between counties. Map 4 (Figure 4) shows that 12 out of 41 EMU CHDIS mobile clinics were offered in places with a medium-to-high percentage (greater than the national average percentage of 8.8%) of individuals with no insurance. Similar patterns were also noted in Map 5 (Figure 5) where 31 out of 41 CHDIS mobile clinics were situated in areas where the percentage of households that have no computer access is above the national average percentage (3.3%), which is often considered a critical barrier for accessing COVID-19 vaccines. As shown in Map 6 (Figure 6), more than 75 percent of EMU CHDIS mobile clinics were offered in communities with medium-to-high SVI (greater than the state average percentage of 0.5). This map also illustrates that socially vulnerable communities are being incorporated into key public health decision-making and activities when EMU CHDIS mobile clinics were set up to allocate, distribute and administer the COVID-19 vaccines.

## 5. Trusted Messengers: Increasing the Availability of COVID-19 Vaccination Sites without Partnering with Trusted Leaders May Not Translate to Accessibility

The EMU CHDIS team identified bilingual community leaders and conducted training to support their roles as trusted messengers with resources and tools that were translated into 14 languages and dialects to educate their respective communities (Table 1). Training included participation in at least one 2-h Zoom education session run by a team Nurse Educator and Nursing and Dietetics faculty members and also on-going technical assistance support. Participants were trained on COVID-19 and influenza vital statistics (e.g., infection rates, death rates, associated health disparities), preventive measures, updated vaccination guidelines, healthy immune system, and communication strategies. These trusted messengers were also present at EMU CHDIS COVID-19 vaccination clinics, which further supported the promotion of COVID-19 vaccine messaging that built vaccine confidence among community members in linguistically and culturally accessible ways.

## 6. Conclusions

As the COVID-19 pandemic progressed, mass vaccination sites at the state-level did not adequately provide access to COVID-19 vaccines to underserved communities; particularly those with the additional barrier of LEP. The current paper described an approach for delivering COVID-19 vaccinations where they were most needed. Utilizing GIS in the planning stages allowed the EMU CHDIS team to identify locations where priority populations with the greatest needs reside and design a vaccine distribution plan suited to effectively reach them. Population data and other key determinants layered under infrastructure data with stakeholders’ input ensured the most accessible sites were chosen. Working with trusted messengers and partnering with community-based organizations, EMU CHDIS mobile clinics were set up at locations where underserved AAs resided and where computer accessibility (or the ability to use a computer for vaccine appointment registration) was lacking. This was accomplished while considering areas of medium-to-high SVI and LEP that hindered registration for vaccinations.

There were several challenges including financial considerations that impeded the expansion of community-based vaccination sites. Additionally, some mobile vaccination sites had limited Wi-Fi hotspots for registration and some had a lack of adequate space to ensure patient privacy. Despite the challenges, the implementation of mobile clinics using GIS maps expanded COVID-19 vaccine access, and community-driven, multi-sector partnerships increased capacity to enhance efforts and facilitate access of COVID-19 vaccinations for hard-to-reach populations. As of today, EMU CHDIS mobile clinics have served more than 3700 hard-to-reach individuals in Michigan by providing access to COVID-19 vaccines.

## Figures and Tables

**Table 1:** Languages of translation for COVID communication messages.

**Asian Languages**
Bangla
Burmese
Chinese
Hmong
Japanese
Korean
Nepali
Punjabi
Tamil
Thai
Urdu
Vietnamese
**Arabic Languages**
Burmese Dialects
Falal
Haka
Tedim

## Data Availability

Data sharing is not applicable to this article.

## References

[1] Office of the Assistant Secretary for Planning and Evaluation (2021). Disparities in COVID-19 Vaccination Rates across Racial and Ethnic Minority Groups in the United States. https://aspe.hhs.gov/sites/default/files/private/pdf/265511/vaccination-disparities-brief.pdf.

[2] Rossen L.M., Branum A.M., Ahmad F.B., Sutton P., Anderson R.N. (2020). Excess Deaths Associated with COVID-19, by Age and Race and Ethnicity—United States, 26 January–3 October 2020. MMWR Morb. Mortal. Wkly. Rep..

[3] Asian & Pacific Islander American Health Forum (2021). Written Statement for the Record for the Hearing Entitled “Road to Recovery: Ramping up COVID-19 Vaccines, Testing and Medical Supply Chain”. https://www.apiahf.org/wp-content/uploads/2021/02/2021_02_03_Testimony_Juliet_K_Choi_APIAHF_COVID_Vaccine_Hearing.pdf.

[4] Cohn D., Passel J.S. (2018). A Record 64 Million Americans Live in Multigenerational Households. https://www.pewresearch.org/fact-tank/2018/04/05/a-record-64-million-americans-live-in-multigenerational-households/.

[5] Dang E., Huang S., Kwok A., Lung H., Park M., Yueh E. (2020). COVID-19 and Advancing Asian American Recovery. https://www.mckinsey.com/~/media/mckinsey/industries/public%20and%20social%20sector/our%20insights/covid%2019%20and%20advancing%20asian%20american%20recovery/covid-19-and-advancing-asian-american-recovery-v3.pdf.

[6] U.S. Census Bureau ACS 5-Year Estimates. MDAT 2019. https://data.census.gov/mdat/#/search?ds=ACSPUMS5Y2019&cv=ENG&wt=PWGTP.

[7] APIA Vote (2020). 2020 State Factsheet: Michigan. https://apiavote.org/wp-content/uploads/Michigan-2020.pdf.

[8] CDC Ensuring Equity in COVID-19 Vaccine Distribution. https://www.hrsa.gov/coronavirus/health-center-program.

[9] Hughes M.M., Wang A., Grossman M.K., Pun E., Whiteman A., Deng L., Hallisey E., Sharpe J.D., Ussery E.N., Stokley S. (2021). County-level COVID-19 vaccination coverage and social vulnerability—United States, 14 December 2020–1 March 2021. MMWR Morb. Mortal. Wkly. Rep..

[10] Bibbins-Domingo K., Petersen M., Havlir D. (2021). Taking vaccine to where the virus is—Equity and effectiveness in coronavirus vaccinations. JAMA Health Forum.

[11] Himmelstein J., Himmelstein D.U., Woolhandler S., Dickman S., Cai C., McCormick D. (2022). COVID-19–Related Care for Hispanic Elderly Adults With Limited English Proficiency. Ann. Intern. Med..

[12] Fuchs J.R., Fuchs J.W., Tietz S.E., Lum H.D. (2021). Older Adults with Limited English Proficiency Need Equitable COVID-19 Vaccine Access. J. Am. Geriatr. Soc..

[13] Ellis N.T. A Vaccination Site Meant to Serve a Hard-Hit Latino Neighborhood in New York instead Serviced More Whites from Other Areas. https://www.cnn.com/2021/01/30/us/new-york-vaccine-disparities/index.html.

[14] Bonner L. For Black, Latinx, Native American Residents, Community Connections Are Key to COVID-19 Vaccination Success. http://www.ncpolicywatch.com/2021/02/19/for-black-latinx-native-american-residents-community-connections-key-to-covid-19-vaccination-success/.

[15] Oteri J., Hussaini M.I., Bawa S., Ibizugbe S., Lambo K., Mogekwu F., Wiwa O., Seaman V., Kolbe-Booysen O., Braka F. (2021). Application of the Geographic Information System (GIS) in immunisation service delivery; its use in the 2017/2018 measles vaccination campaign in Nigeria. Vaccine.

[16] Ali D., Levin A., Abdulkarim M., Tijjani U., Ahmed B., Namalam F., Oyewole F., Dougherty L. (2020). A cost-effectiveness analysis of traditional and geographic information system-supported microplanning approaches for routine immunization program management in northern Nigeria. Vaccine.

[17] Kazi A.M., Ali M., Ayub K., Kalimuddin H., Zubair K., Kazi A.N., Artani A., Ali S.A. (2017). Geo-spatial reporting for monitoring of household immunization coverage through mobile phones: Findings from a feasibility study. Int. J. Med. Inform..

[18] Utazi C.E., Thorley J., Alegana V.A., Ferrari M.J., Takahashi S., Metcalf C.J.E., Lessler J., Cutts F.T., Tatem A.J. (2019). Mapping vaccination coverage to explore the effects of delivery mechanisms and inform vaccination strategies. Nat. Commun..

[19] U.S. Census Bureau TIGER Data Products. https://www.census.gov/tiger-data.

[20] GISCorps Hundreds of Volunteers Create Nationwide COVID-19 Testing and Vaccination Site Layers. https://www.giscorps.org/covid19-esri-282/.

[21] State of Michigan COVID 19 Dashboard. https://www.michigan.gov/coronavirus/resources/covid-19-vaccine/covid-19-dashboard.

[22] U.S. Census Bureau American Community Survey 2016–2020 5-Year Data Release. https://www.census.gov/newsroom/press-kits/2021/acs-5-year.html.

[23] Agency for Toxic Substances and Disease Registry CDC/ATSDR SVI Data and Documentation Download, Place and Health, ATSDR. https://www.atsdr.cdc.gov/placeandhealth/svi/data_documentation_download.html.

[24] Environmental Systems Research Institute (ESRI) (2021). ArcGIS Pro. https://www.esri.com/en-us/arcgis/products/arcgis-pro/overview.

[25] ArcGIS—EMU CHDIS Vaccine Project. https://arcg.is/0OSrWq.

[26] CDC/ATSDR Social Vulnerability Index. https://www.atsdr.cdc.gov/placeandhealth/svi/index.html.

